# War Savings Associations: The Action of Two Great Hospitals

**Published:** 1917-02-24

**Authors:** 


### WAR SAVINGS ASSOCIATIONS.

### The Action of Two Great Hospitals.

Mr. R. M. Kindersley, the Chairman of the War Savings Committee, in a letter to the *Times,* dated February 2, states that there are in existence over 20,000 War Savings Associations, with a membership of some millions, who are contributing weekly sums of 6d. and upwards towards the purchase of War Savings Certificates, and that these Associations are increasing at the rate of 700 to 900 per week.

So thoroughly has the issue of the War Savings Certificates been advertised that it is surprising that the advantages and object of the scheme are not understood by most people. Yet a curious incident cropped up the other day in connection with an Association started by the Council of a municipality. A number of subscribers, having saved long enough to become entitled to their first certificates, on being handed these in exchange for their cards, regarded them with surprise, and asked what they meant. On being informed that they represented a receipt for 15s. 6d., which, in course of time, would be worth £1, they were still more surprised as they were under the impression that they were *giving* their sixpences to the Government for the purpose of carrying on the war and beating the Germans; some, indeed, appeared to be more or less disappointed that it was a case of *lending* and not giving.

The fact that there is a good opening for Savings Associations at the hospitals has been recognised in several places, notably at St. Thomas's and Guy's, where the movement has become firmly established. Permanently employed male officers and servants are at present, by the nature of things, few in number, and, therefore, the bulk of the membership at hospitals is to be found amongst the women.

### St. Thomas's Hospital.

At St. Thomas's a War Savings Association has now been running for eight months, having been started on June 28, 1916, and although the staff of men are encouraged to save, they do not come within the scheme, which is managed by women for women. There are now 337 members, and up to the end of 1916, 607 certificates were purchased, a total which has risen now to 939. This may be described as a very creditable record for six months' work. The Association is managed by a committee of which the matron and secretary are members, but the actual work is done by two or three of the leading sisters, especially active being the sister of the Preliminary Training School, while a sister in the matron's office acts as treasurer. The method of work is that all cash received is as quickly as possible turned into Savings Certificates, and the dates registered; thus the smallest subscriber gets interest on the investment from the commencement. When a nurse, scrubber, or other member has subscribed sufficient she gets a certificate from an older or newer bundle according to the length of time for which she has been a subscriber. In this way the member who has been paying for some time gets an advantage over the one who puts down 15s. 6d. in one lump. This method, which is a very fair one, was considered by the committee to be better than the one prevailing in some other Associations, where priority of certificates is decided by draw.

All honour is due to the sisters of St. Thomas's for taking up the scheme so vigorously, and their action, which it is understood was the first taken in the hospital world, has encouraged others to embark in the same provident and patriotic undertaking.

### Guy's Hospital.

Quickly following the good example set by St. Thomas's, the Guy's Hospital Association was started on August 1. 1916, by means of a general meeting, advertised by posters, attended by some fifty or sixty people. Officers and committees, representatives of all classes of employees—viz. works, medical school, nurses, administrative workers, etc.—were appointed, and committee members undertook the collection of subscriptions in their own departments. Cash is handed in to the secretary of the Association every Saturday, when books are balanced and certificates bought and handed to the treasurer for safe custody. When a member becomes eligible for a certificate he receives a letter stating the fact, on the authority of which the treasurer hands him his certificate. When members tie for priority in receiving certificates their names are taken in alphabetical order, but little hardship arises as there is not often more than a week's difference in the date of issue. The scheme is working well and gets stronger every week.

### Associations at Other Hospitals.

We understand that there is a flourishing Association at the Evelina Hospital for Children, and that other hospitals in London and the provinces are taking part in the movement. We shall be glad to publish the names of these hospitals if the hon. secretaries of the Associations will kindly send particulars. For printed matter, including suggestions of model schemes, applications should be made to the National War Savings Committee, 18 and 19 Abingdon Street, Westminster, S.W.

